# Preparedness and management during the first phase of the COVID-19 outbreak - a survey among emergency primary care services in Norway

**DOI:** 10.1186/s12913-022-08284-9

**Published:** 2022-07-11

**Authors:** Jonas Nordvik Dale, Tone Morken, Knut Eirik Eliassen, Jesper Blinkenberg, Guri Rørtveit, Steinar Hunskaar, Ingrid Keilegavlen Rebnord, Valborg Baste

**Affiliations:** 1 National Centre for Emergency Primary Health Care, NORCE Norwegian Research Centre, Bergen, Norway; 2 Department of Global Public Health and Primary Care, University of Bergen, Bergen, Norway; 3 Research Unit for General Practice, NORCE Norwegian Research Centre, Bergen, Norway

**Keywords:** Primary care, COVID-19, Pandemic, Primary care clinic, Out of hours service, Emergency primary care, Preparedness

## Abstract

**Background:**

The emergency primary care (EPC) services in Norway have been at the frontline of the COVID-19 pandemic. Knowledge about the EPC services’ management of the COVID-19 outbreak can be used to prepare for future outbreaks and improve patient management. The objectives of this study were to identify pandemic preparedness and management strategies in EPC centres in Norway during the COVID-19 outbreak.

**Methods:**

Questions regarding patient management of the COVID-19 outbreak were included in data collection for the National Out-Of-Hours Services Registry. The data collection was web-based, and an invitation was sent by email to the managers of all EPC services in Norway in June 2020. The EPC services were asked questions about pre-pandemic preparedness, access to personal protective equipment (PPE), organizational measures taken, and how staffing was organized during the onset of the pandemic.

**Results:**

There were 169 municipal and inter-municipal EPC services in Norway in 2020, and all responded to the questionnaire. Among the EPC services, 66.7% (*n* = 112) had a pandemic plan, but only 4.2% had performed training for pandemic preparedness. Further, fewer than half of the EPC centres (47.5%) had access to supplies of PPE, and 92.8% answered that they needed extra supplies of PPE. 75.3% of the EPC services established one or more respiratory clinics. Staffing with other personnel than usual was done in 44.6% (*n* = 74) of the EPC services. All EPC services except one implemented new strategies for assessing patients, while about half of the wards implemented new strategies for responding to emergency calls.

None of the largest EPC services experienced that their pandemic plan was adequate, while 13.3% of the medium-sized EPC services and 48.9% of the small EPC services reported having an adequate pandemic plan.

**Conclusions:**

Even though the EPC services lacked well-tested plans and had insufficient supplies of PPE at the outbreak of the COVID-19 pandemic, most services adapted to the pandemic by altering the ways they worked and by hiring health care professionals from other disciplines. These observations may help decision makers plan for future pandemics.

**Supplementary Information:**

The online version contains supplementary material available at 10.1186/s12913-022-08284-9.

## Background

Primary care is at the very frontline of the COVID-19 pandemic, and the outbreak has had a huge toll on primary care systems [1]. The consequences of a global outbreak on the Norwegian primary care system have previously been demonstrated for the influenza pandemic in 2009 [2, 3]. The SARS-CoV-2-virus, with its ability to live longer and extend the duration of illness compared with other viral infections such as the viruses that cause seasonal flu, has a much higher transmission rate than most other viral respiratory diseases [4, 5]. A rapidly spreading infectious disease with a risk of severe outcome in parts of the population can overwhelm health care systems resulting in a devastating situation. With the COVID-19 pandemic, this was especially true in Italy in the initial phase [6]. Other countries so far (as of 2021) have fared better, such as many of the Scandinavian countries [7], Australia, and South Korea. Tools such as lockdowns, disease tracing, and quarantining prevent the spread of the disease, but at substantial costs for society. Reorganizing primary health care has been necessary, with various solutions in different countries [8].

Most patients with COVID-19 experience mild to moderate symptoms. This means that most medical care will be provided by primary health care providers in places where a primary care system is well developed [9]. Countries with a strong primary care system may respond effectively to an epidemic outbreak [10, 11] and also reduce unnecessary hospital admissions [12].

In Norway, the municipalities are legally responsible for primary care and 24/7 emergency medical services for all inhabitants [13], and general practitioners (GPs) function as gatekeepers for referrals to secondary care. The differences between rural and urban municipalities are large. Differences include population, area, population density, economics, use of primary health care services, and proximity to specialized health care services.. The smallest municipality in Norway covers only 198 inhabitants, while the largest covers approximately 630,000 inhabitants. Out of hours, the emergency primary care (EPC) services are the first point of contact with the Norwegian medical system for all inhabitants. Each EPC service can cover one or more municipalities based on local priorities. Understanding how the EPC services have been organized to meet the demands of the pandemic is therefore a key part in understanding primary care pandemic management. Norway has had relatively few COVID-19 cases as of January 2022, approximately 7900 cases per 100,000 inhabitants, including 136 hospitalized and 25 deaths per 100,000 [14]. The relatively small infection rates in Norway in 2020 could indicate an efficient initial pandemic strategy as infection levels stayed low throughout 2020 [15], and experiences from Norway might be useful in planning for pandemic preparedness, also elsewhere, in the future.

For primary care to meet any pandemic effectively, evidence-based knowledge is necessary regarding plans to handle an overwhelming number of patients, the organization of units to avoid transmission to health care workers and other patients, and the ability of public health systems to perform testing, tracing, and isolation. Although examples of how the COVID-19-pandemic was managed in primary care in a Norwegian municipality [16] and GPs’ offices [17] have been described, it is not known how the pandemic was managed throughout the country.

The objectives of this study were 1) to identify pandemic preparedness and management strategies in EPC services in Norway during the COVID-19 pandemic, 2) to investigate potential differences in preparedness and patient management between EPC centres covering various population sizes, and 3) to summarize the experiences and provide evidence for decision makers for future pandemic planning.

## Methods

This cross-sectional study was based on a survey among the 169 EPC services in Norway and was part of the biennial data collection for the National Out-Of-Hours Services Registry (NOOHR). The EPC services cover one or more municipalities, including those with fewer than one thousand inhabitants to those with several hundreds of thousands of inhabitants. In this text, EPC services refer to the organisational units that provide out-of-hours primary care services in Norway. The National Centre for Emergency Primary Health Care sent a web-based questionnaire by email to the manager of each EPC service in Norway in June 2020. The NOOHR is maintained by request from The Norwegian Ministry of Health and Care Services. Non-responders received a reminder email one and two weeks after the first email. Subsequently, EPC services that still had not responded were contacted by telephone. The survey was approved by the Norwegian Centre for Research Data (project 13,326, 2020). All methods were carried out in accordance with relevant guidelines and regulations as given by the Declaration of Helsinki.

The current survey included several COVID-19-related questions, and the EPC services answered based on their experiences with the pandemic from March 2020 to June 2020. Appendix A shows the full text questions with short phrases and answer options. The questions were grouped into three study targets.

1. **Pandemic preparedness** before the outbreak was investigated with questions about any ‘Pandemic response plan’ with a follow-up question: Was the plan adequate for meeting the COVID-19 outbreak? Further, we asked about ‘Pandemic training’, ‘Access to personal protective equipment (PPE)’ and ‘Ordering additional PPE’. For the last question there was a follow-up question: Did the EPC get the needed supplies? The answer options for these questions were yes/no/do not know (Appendix A).
2. **Organization and staffing** included questions regarding how the EPC reorganized and staffed itself to handle infectious patients and about the municipalities’ plans for treatment and follow up of COVID-19 patients. We asked whether the EPC services established a ‘Separate infection room in the EPC service’ and whether the EPC services had to use other personnel than normal to staff the regular EPC service, and if so why this was necessary (Appendix A). We asked whether the EPC services established a ‘Separate airway clinic’ and what kind of personnel worked at this clinic (‘Airway clinic personnel’). By airway clinics we refer to an organizational structure specifically designed as the first pass for the suspected patients who attend the EPC with fever or respiratory symptoms. Ideally, the airway clinics are stand-alone units with clear pathways of entry and exit with reduced chance for mixing patients, relatives, and health care staff. Further questions were asked about whether the EPC services used ‘GP extraordinary working in the EPC services’, whether the municipality created ‘COVID-19 wards’, and a question regarding the creation of a team of health care professionals working for the municipality with COVID-19-specific tasks such as infection tracing and advising the general public as outlined by the Norwegian Institute of Public Health (‘Independent quarantine teams’) [18]. The answer options for these questions were yes/no/do not know.
3. **Patient management** included questions concerning how the patient was assessed by the EPC services. We asked about ‘New strategies for assessing EPC patients’, with several answers possible (Appendix A), and we asked about ‘New strategies for responding to emergency calls’ with answer options of yes/no/do not know.

We also asked about changes in procedures concerning referrals to the hospital in terms of ‘New written procedures for referrals to secondary health care services’ and ‘Obligation to discuss same-day referrals with the doctor at the local hospital’ with answer options yes/no and an option to describe more if ‘yes’ was selected.

The total number of inhabitants covered by each EPC service was gathered from Statistics Norway. The EPC services were divided into three groups based on the population each EPC had responsibility for, namely small (< 10,000), medium (10,000–99,999), and large (> 100,000).

### Analyses

Percentages and 95% confidence intervals (CIs) were calculated for all the survey questions to describe the distributions. A chi-square test was applied to test for differences in pandemic preparedness and in measures taken to meet the demands of the pandemic between the EPC services by population size. Some of the questions were not answered by the services (between 1 and 5 services) and were thereby not included in the analysis for the specific question. A significance level of α = 0.05 was used. All statistical analyses were performed in SPSS (Statistical Product and Service Solutions, version 27.0.1 for Windows,©SPSS Inc. 1989–2020).

## Results

The questionnaire had a 100% response rate, and the data covered all 169 municipal and inter-municipal EPC centres in Norway in 2020. There were 82 small, 76 medium, and 11 large EPC centres covering a mean number of inhabitants of 4275 (range: 435–9892) for small EPCs, 33,821 (range: 10,084 - 94,875) for medium EPCs and 206,309 (range: 101,248 – 693,949) for large EPCs. The 11 largest EPC centres covered approximately 2,3 million (43%) of Norway’s 5,4 million inhabitants.

### Pandemic preparedness among EPC services before the outbreak

Before the COVID-19 outbreak, 66.7% (*n* = 112) of the EPC services had a pandemic plan (Table 1). Among these, 25.0% answered that the pandemic plan was adequate for meeting the outbreak. There were differences between the EPC services by population size, and none of the largest EPC services experienced that the pandemic plan was adequate, while 13.3% (95% CI 3.0–23.7) of the medium-sized EPC services and 48.9% (95% CI 33.7–64.1) of the small EPC services had an adequate pandemic plan (*p* ≤ 0.001). Team training for pandemic preparedness had been performed in 4.2% of the EPC services. Among all EPC services, 47.5% reported having access to stored PPE for a pandemic. A total of 92.8% (*n* = 155) of the EPC services had to order additional PPE, and 96.8% of these reported that they got the needed supplies. There were no significant differences between the EPC services by population size in terms of access to stored PPE or a need to order PPE at the breakout of the pandemic.

**Table 1:** Preparedness among emergency primary care (EPC) services before the COVID-19 outbreak (*n* = 169)

Pandemic preparedness	Inhabitants covered by the EPC services								
	< 10,000 (*n* = 76)		10,000–99,999 (*n* = 82)		≥ 100,000 (*n* = 11)		Total		*p*-value
	n	% [95% CI]	n	% [95% CI]	n	% [95% CI]	n	% [95% CI]	
Pandemic response plan	51	68.0 [57.2; 78.8]	54	65.9 [55.3; 76.3]	7	63.6 [29.7; 97.5]	112	66.7 [59.5; 73.9]	< 0.001
Pandemic training	3	4.0 [−0.5; 8.5]	3	1.2 [−1.2; 3.7]	3	27.3 [−4.1; 58.6]	9	4.2 [1.1; 7.3]	< 0.001
Access to PPE	39	54.9 [43.1; 66.8]	33	42.3 [31.1; 53.5]	4	36.4 [2.5; 70.3]	76	47.5 [39.7; 55.3]	0.227
Order additional PPE	66	88.0 [80.5; 95.5]	78	96.3 [92.1; 100]	11	100 [100; 100]	155	92.8 [88.9; 96.8]	0.085

*EPC* Emergency primary care, *PPE* Personal protective equipment, *CI* Confidence interval

### Organization and staffing during the pandemic outbreak among EPC services

Separate infection rooms to examine patients with suspected COVID-19 were established in nearly all EPC services (92.8%) (Table 2). Maintaining satisfactory services at the EPC services required more health care professionals than usual. Personnel from outside the regular staff of. EPC nurses, EPC doctors, and GPs) were needed in 44.6% (*n* = 74) of the EPC services during the outbreak, including 72.7% of the large, 57.5% of the medium, and 26.7% of the small EPC services (*p* ≤ 0.001). Among the EPC services that hired staff outside their normal recruitment areas, the following causes were reported: Increased workload at the EPC services (70%, *n* = 52), staffing of the airway clinic (54%, *n* = 40), quarantine of EPC services personnel (24%, *n* = 18), and sickness among EPC services personnel (11%, *n* = 8). The largest EPC services were more likely to hire students, school nurses, medical secretaries, and other types of health care professionals to staff both the normal EPC services and the airway clinics compared with the smaller EPC services (*p* ≤ 0.001).

**Table 2:** Organization and staffing among emergency primary care (EPC) services at the COVID-19 pandemic outbreak in March–June 2020 (n = 169)

Organization and staffing	Inhabitants covered by the EPC service								
	< 10,000 (*n* = 76)		10,000–99,999 (*n* = 82)		≥ 100,000 (*n* = 11)		Total		*p*-value
	n	% [95% CI]	n	% [95% CI]	n	% [95% CI]	N	%	
Separate infection ward in EPC service	69	92.0 [85.7; 98.3]	75	92.6 [86.8; 98.4]	11	100 [100; 100]	155	92.8 [88.9; 96.8]	0.627
EPC clinic staffed by non-EPC personnel	20	26.7 [16.4; 36.9]	46	57.5 [46.4; 68.6]	8	72.7 [41.4; 104.1]	74	44.6 [36.9; 52.2]	< 0.001
GPs extraordinary working in the EPC service	3	4.0 [−0.5; 8.5]	19	23.8 [14.2; 33.3]	2	18.2 [−9.0; 45.4]	24	14.5 [9.1; 19.9]	0.002
COVID-19 wards	61	81.3 [72.3; 90.4]	70	86.4 [78.8; 94.0]	11	100 [100; 100]	142	85.0 [79.6; 90.5]	0.239
Independent quarantine team	46	62.2 [50.9; 73.5]	65	82.3 [73.7; 90.9]	10	90.9 [70.7; 111.2]	121	73.8 [67.0; 80.6]	0.008
Separate airway clinic	46	62.2 [58.9; 73.5]	69	85.2 [77.3; 93.1]	10	90.9 [70.7; 111.2]	125	75.3 [68.7; 81.9]	0.002
Airway clinic staffed by non-EPC personnel	31	40.8 [29.5; 52.1]	54	65.9 [55.4; 76.3]	10	90.9 [70.7; 111.2]	95	56.2 [48.7; 63.8]	< 0.001

One or more airway clinics were established in 75.3% of the EPC services. These airway clinics were created in 90.9% (95% CI 70.7–111.2) of the largest, 85.2% (95% CI 77.3–93.1) of the medium, and 62.2% (95% CI 58.9–73.5) of the smallest EPC services (*p* = 0.002). Among the EPC services with airway clinics (75.3%), the most frequently used personnel were GPs, EPC doctors, and EPC nurses (Table 3). To staff the airway clinics, 56.2% of the EPC services had to hire personnel from outside their normal recruiting areas, including 90.9% of the largest 65.9% of the medium, and 40.8% of the smallest EPC services, (*p* ≤ 0.001).

**Table 3:** Staffing of airway clinics during the COVID-19 pandemic outbreak in March–June 2020 (several answers possible) (*n* = 125)

Staffing of airway clinics	Inhabitants covered by the EPC service							
	< 10,000 (*n* = 76)		10,000–99,999 (*n* = 82)		≥ 100,000 (*n* = 11)		Total	
	n	%	n	%	n	%	n	%
General practitioners	31	40.7	55	67.0	8	72.7	94	55.6
Emergency primary care doctors	28	36.8	43	52.4	6	54.5	77	45.5
Emergency primary care nurses	23	30.2	47	57.3	5	45.4	75	44.3
Personnel from other parts of the primary health care	9	11.8	31	37.8	8	72.7	48	28.4
School nurse	5	6.5	27	32.9	5	45.4	37	21.8
Students	4	5.2	22	26.8	3	27.2	29	17.1
Others (e.g., medical secretaries)	19	25.0	22	26.8	6	54.5	47	27.8

In 14.5% of the EPC services, GPs who did not normally participate in the EPC service attended the on-call scheme due to the outbreak. A total of 85.0% of the EPC services reported that the municipality had established local municipal emergency beds to treat COVID-19 patients. Among these, 59.0% reported that the local hospital beds were established at a nursing home, and 23.0% reported that the hospital beds were established at an emergency hospital in the municipality. Independent quarantine teams were established in 73.8% of the EPC services.

### Patient management

New strategies for assessing patients with symptoms indicative of COVID-19 were implemented in 99.4% of the EPC services, while 52.4% implemented new strategies for responding to emergency calls (Table 4). Telephone consultation was established as a strategy for patient management in 85.2% (*n* = 144) of the EPC services, and 60.4% (*n* = 102) implemented video consultations. Procedures for patients waiting in their cars instead of a waiting room were established in 84.0% (*n* = 142) of the EPC services, and 62.7% (*n* = 106) performed the clinical assessment of patients in their cars.

**Table 4:** Patient management in the emergency primary care (EPC) services during the COVID-19 pandemic outbreak in March–June 2020 (*n* = 169)

Patient management	Inhabitants covered by the EPC service								
	< 10,000 (*n* = 76)		10,000–99,999 (*n* = 82)		≥ 100,000 (*n* = 11)		Total		*p*-value
	n	% [95% CI]	n	% [95% CI]	n	% [95% CI]	n	%	
New strategies for assessing EPC patients	75	98.7 [96.1; 101.3]	82	100 [100; 100]	11	100 [100; 100]	168	99.4 [98.2; 100.6]	0.540
New strategies for responding to emergency calls	43	58.1 [46.6; 69.6]	36	45.6 [34.4; 56.8]	7	63.6 [29.7; 97.5]	86	52.4 [44.7; 60.2]	0.223

The hospital had given written guidelines on hospital admissions during the pandemic outbreak to 82.2% (*n* = 139) of the EPC services. Also, 67.6% (*n* = 94) of these EPC services reported that the EPC doctor was obliged to discuss admissions with the local hospital, in contrast to pre-pandemic routines.

## Discussion

We found that two thirds of the EPC services had a pandemic plan prior to the COVID-19 outbreak, but only a quarter of these plans were considered adequate for the outbreak. Few EPC services had performed team training for a pandemic outbreak, and fewer than half of the EPC services had adequate access to PPE at the beginning of the pandemic. Separate infection rooms in the EPC to assess patients with suspected COVID-19 and separate airways clinics were the most commonly used measures taken to redirect the flow of patients with suspected COVID-19 away from the regular EPC services.

The smaller EPC services less often created separate infection rooms airway clinics or hired personnel from outside the normal EPC staff, although even the smallest EPC services used multiple new approaches in patient management. The largest EPC services were significantly more likely to create airway clinics and to staff these with non-EPC personnel.

### Pandemic preparedness among EPC services before the outbreak

Only two thirds of the EPCs had a pandemic plan, and almost none (4.2%) of the EPC services had a functional pandemic response plan that they had tested and/or trained with prior to the COVID-19 pandemic. This lack of pandemic preparedness and planning has been addressed elsewhere [19, 20] and a North American paper from 2017 underlines the importance of pre-disaster training amongst public health care workers [4]. In Norway, the municipalities are obliged to develop plans describing their strategies for responding to a pandemic [21], while training for a pandemic-level event is not obligatory. The deficient pandemic planning and training described in our study points to areas that could benefit from more scrutiny by decision makers in the future especially since pandemic planning is part of the law.

Insufficient pandemic plans for Norway were also observed after the influenza pandemic in 2009 [22], where the Norwegian government pointed to several areas in need of improvement to handle future pandemics. The government reported that more robust pandemic plans with differentiated plans for different phases of a pandemic was important, as well as a better understanding of how personnel in the primary health care services interact and are utilized during a pandemic. The WHO in their post-2009 influenza pandemic report [23] summarized 15 recommendations to strengthen pandemic preparedness. Amongst these recommendations were a need for better pandemic planning and strengthening of the public health workforce.

Both nationally and internationally the importance of pandemic planning and stable access to qualified health care personnel has been shown. Our study indicates plans for sufficient staffing should have been better, and we point to areas that could have fared better if previous experiences had been taken into consideration.

Preparing for an influenza pandemic is “a continuous process of planning, exercising, revising and translating into action, national and subnational pandemic preparedness and response plans” [24]. In many countries, the primary health care system is a crucial part of the preparedness and response [9, 25–27]. The importance of training for pandemic preparedness among health care providers was emphasized in China during the COVID-19 crisis [28], the response to which was characterized by high workloads, undertrained staff, and high transmission rates among health care workers as a consequence of not being prepared for a pandemic-level event. The impact of the lack of pre-pandemic planning and training was probably lessened by the fact that the Norwegian government quickly developed guidelines concerning COVID-19 management for the health care services in Norway. Some of these guidelines were also added as temporary laws to further strengthen the flexibility and responsibilities of the municipalities, a course of action previously taken by the Norwegian government during the 2009 influenza pandemic [22].

We found that fewer than half of all EPC services had access to sufficient PPE at the start of the pandemic, and 92.8% of EPC services needed additional PPE during the first few months of the pandemic. This could have led to higher rates of infection among health care workers than what was observed in the general population in Norway [14]. Adequate PPE is crucial to protect both health workers and patients [26, 29], and recommendations for PPE among health personnel in primary care have been presented by the Norwegian Directorate of Health [30]. In a Mexican paper from 2009 [31] it was noted that stockpiling supplies of antiviral medication and PPE was an important part of limiting the negative consequences of the 2009 influenza pandemic. At the start of the COVID-19 pandemic, worries over a lack of PPE supplies were reported, and frontline health care workers needed more gloves, masks, face shields, and gowns than what was stored [9, 32]. Our study indicates that the concern for lack of PPE supplies were partly mitigated by limiting health care workers’ exposure to patients with potential COVID-19 by changing the ways to assess patients, thereby limiting the need for PPE equipment. The effect of such an adaptation is outside the scope of our study. It could be argued that access to sufficient storage of PPE equipment is more important than changing ways to assess patients, which may not always be feasible, and should be prioritized for future pandemics.

### Organization and staffing during the pandemic outbreak among EPC services

Our data show that 75.3% of the EPC services created a separate airway clinic, and almost all EPC services (92.8%) created a separate infection ward in the EPC. This helped create efficient ways to examine and treat patients potentially infected with COVID-19 and reduced the number of contact points these patients had with the health care services. The airway clinics were also an efficient way of distributing limited supplies of PPE to where they were most needed. Airway, respiratory, or fever clinics have been established in primary health care both in Norway and elsewhere in the world as part of the primary COVID-19 care response [9, 26] and have also been used in hospitals in China during the COVID-19 outbreak [33]. Airway clinics in primary health care have been recommended by the Norwegian Association of General Practitioners since the beginning of the pandemic [34].

We found that 44.6% of the EPC services were staffed by other personnel than usual during the outbreak. This especially holds true for the largest EPC services, where there may be greater availability of health care students and health care professionals not working in clinical fields. Increased workload on the EPC, staffing of airways clinics, and the absence of staff due to their own sickness or quarantine were the most common causes for non-EPC personnel to be working at the EPC services during the pandemic outbreak. A Dutch study from the emergency departments in the Netherlands [35] showed a similar shift of nursing personnel from their normal departments to the emergency department to help cope with the increased workload.

Our study is in line with other studies [35, 36] demonstrating health care workers ability to take on different roles when the demand for certain health care services is high. A high degree of work task flexibility among health care workers may be an important part of successful pandemic management and could be a learning point for decision makers in the future.

GPs, EPC doctors, and EPC nurses were the three most common health care workers to staff airways clinics. This means that for many of the EPC services, the primary way to examine patients with suspected COVID-19 symptoms was in an airway clinic staffed with personnel who usually work at the EPC. Though it was not shown in this study, it may be fair to assume that in many cases this resulted in EPC personnel working more than usual, as demonstrated by others [37].

### Patient management

New strategies for assessing patients with symptoms indicative of COVID-19 were implemented in all but one of the EPC services in Norway, while about half implemented new strategies for responding to emergency calls.

Both consultations by phone and video were widely used in the Norwegian EPCs as an alternative way to assess patients. This was probably an important measure in limiting contacts with potential COVID-19 patients and helped limit the use of PPE. The COVID-19 pandemic has pushed health systems to use telemedicine both in primary care in Norway and around the world [1, 38–40]. A switch from physical to video consultation when regarded as safe, was recommended by the Norwegian authorities during the pandemic [41].

The safety of these telemedicine measures, and applicability for different types of consultations vary. Technical issues must be considered. As suggested by others [42], telemedicine might be most suited for simple problems not requiring physical examination. Despite these issues, patient satisfaction with telemedicine has been found to be good [43] and the rate of e-consultations in Norway has expanded during the pandemic.

Despite little pre-pandemic planning and training, the EPC services in Norway adapted to the new situation and managed to establish new ways to treat and handle patients, reorganize core services, and recruit staff to meet the increased workload all within a short time frame while still managing to perform their core tasks. A European study of primary health care services showed the rapid adaptations made to meet the demands of the COVID-19 pandemic [44], and a study from Italy showed the usefulness of rapid and coordinated reorganization of both primary and secondary care during the COVID-19 outbreak [45].

### EPC services - differences by population size

The largest EPC services were significantly more likely to create airway clinics and to staff these with non-EPC personnel. The largest EPCs were also more likely to have a pandemic plan and to have implemented changes, but they were also more likely to report that their plans prior to the pandemic were not sufficient to describe all measures that needed to be taken.

The stratification of the EPC centres into small, medium, and large were based on differences dude to size that might impact our findings. We consider small EPCs to be the most vulnerable as these have the most limited resources available, both financially, structurally and in terms of staff. The largest EPCs covers the largest cities, are more robust and have a much higher patient turnover and buffer capacity in their services. The differences between small and large EPC services may be explained by higher transmission rates in metropolitan areas and a subsequent higher demand for specialized primary health care services and thus for more available personnel. This last point was in part supported by the results of this study, where larger EPC services to a greater degree employed students, school nurses, medical secretaries, and other types of health care professionals to staff both the normal EPC services and the airway clinics. The differences could also be explained by the fact that the smallest EPC services in Norway cover only a few thousand inhabitants, and thus the need for specialized primary health care services are more limited. Several of the smallest municipalities also had zero transmission rates for COVID-19.

The consequences of the observed differences in how the size of the EPC impacts on the level of preparedness and measures taken to handle patient flow during the COVID-19 pandemic were outside the scope of this study. However, management decentralization that enables fast and effective local changes to respond to the evolving pandemic as presented by Ohrling et al. [45] may be an important factor and might hold true for smaller EPC services with shorter “command lines”. Decentralization can help local EPC managers adapt broader national and international guidelines to fit local needs and limitations. One could speculate that some of the differences we observed in our study such as need for additional workers form outside the normal recruitment zones reflect differences in EPC sizes rather than in pandemic preparedness and willingness to adapt. A study from 2021 [46] looked at preparedness in academic emergency departments in India before the COVID-19 pandemic and concluded that pandemic preparedness needs to be assessed according to local needs and available resources. This could support our reasoning with respect to the observed differences between differently sized EPCs in Norway. This would indicate that decision makers need to take variance of size into consideration when updating pandemic plans.

### Strengths and limitations

The data gathered for this study cover every *municipality* in Norway. All EPC services answered the survey, giving a unique overview of how the primary care emergency medical services responded to the COVID-19 pandemic. Although some of the answers were incomplete, this only applied to between 1 and 5 services for the different questions and most likely did not have a significant impact on the overall results.

The survey covered the first three months of the pandemic outbreak, and the results cannot be transferred to how the municipalities and EPCs functioned later during the pandemic. The study instead gives an indication of the organization prior to the outbreak and the changes undertaken in the initial stages of the pandemic.

A limitation of the study is the survey participants. The questionnaire was sent out to the manager of each EPC centre, but some questions were targeted at the municipality and not the EPC, which could lead to some answers having low validity. However, most of the questions pertaining to the main study goals were directly related to the EPC centres that answered the questionnaire.

## Conclusions

Even though the EPC services lacked well-tested plans and had insufficient storage of PPE equipment at the outbreak of the COVID-19 pandemic, most services adapted to the pandemic by altering the way they worked and by hiring health care professionals from other disciplines. These observations may help decision makers plan for future pandemics.

## Supplementary Information

**Additional file 1.**

## Data Availability

The datasets generated and analysed during the current study are not publicly available due to personal data in the dataset but are available from the corresponding author on reasonable request.

## Abbreviations

ECP: Emergency primary care
PPE: Personal protective equipment
CI: Confidence interval
GP: General practitioner
NOOHR: National Out-Of-Hours Services Registry
