# Supplementary material for: Preparedness and management during the first phase of the COVID-19 outbreak - a survey among emergency primary care services in Norway
Source: BMC Health Serv Res. 2022 Jul 11;22:896. doi: 10.1186/s12913-022-08284-9 (PMC9275270; doi:10.1186/s12913-022-08284-9)
Supplement: Supplementary file 1 — Additional file 1. [file 12913_2022_8284_MOESM1_ESM.docx]

Appendix A – COVID-19–related questions from the National Out-Of-Hours Services Registry on organizational data for EPC services in Norway

|  |  |  |  |
| --- | --- | --- | --- |
|  | Question | Short phrase | Answer options |
| Pandemic preparedness | |  |  |
|  | Did the EPC* service or municipality(ies) covered by the EPC service have a pandemic response plan prior to the COVID-19 pandemic? | Pandemic response plan | Yes/ No/ Don`t know |
|  | - *Was the plan adequate for meeting the covid-19 outbreak* |  | Yes/ No/ Don`t know |
|  |  |  |  |
|  | Did the EPC service participate in pandemic training prior the COVID-19 pandemic | Pandemic training | Yes/ No/ Don`t know |
|  |  |  |  |
|  | Did the EPC service have access to stored PPE** equipment for use in the event of a pandemic event? | Access to PPE | Yes/ No/ Don`t know |
|  |  |  |  |
|  | Did the EPC service have to order additional supplies of PPE to meet the demand during the period of March–June 2020 | Order additional PPE | Yes/ No |
|  | - *Did the EPC service get the needed supplies* |  | Yes/ No/ Don`t know |
|  |  |  |  |
| Organization and staffing | |  |  |
|  | Did the EPC service establish a separate infection room for the examination of patients potentially infected with COVID-19 in March–June 2020 | Separate infection room in the EPC service | Yes/ No |
|  |  |  |  |
|  | Was there a need for personnel from outside the EPC service to staff the regular EPC service | EPC service staffing | Yes/ No |
|  |  |  |  |
|  | - *If other personnel were used, why was this necessary?* |  | - Regular EPC personnel in quarantine - Regular EPC personnel sick - Regular personnel in an at-risk group and not able to work - Increased workload - EPC personnel used in other pandemic-related activities in the municipality - Other |
|  |  |  |  |
|  |  |  |  |
|  | Did GPs not normally participating in on-call duties at the EPC service participate because of the pandemic? | GPs extraordinary working in the EPC service | Yes/ No |
|  |  |  |  |
|  | Did the municipality(ies) of the EPC service create COVID-19 wards capable of monitoring and treating patients infected with COVID-19? | COVID-19 wards | Yes/ No/ Don`t know |
|  | Did the municipality(ies) covered by the EPC service create an independent quarantine team to handle infection control on an individual level during the pandemic? | Independent quarantine team | Yes/ No |
|  |  |  |  |
|  | Did the EPC service establish separate airway clinics? | Separate airway clinic | Yes/ No. If yes, state number of clinics |
|  |  |  |  |
|  | What type of personnel staffed the airway clinic in March–June 2020? (multiple answers possible) | Airway clinic personnel | - EPC nurses - EPC doctors - GPs - School nurses - Personnel from other parts of the primary health care - Students - Cleaners - Others (e.g., medical secretaries) |
|  |  |  |  |
| Patient management | |  |  |
|  | Did the EPC service use any of the following strategies to assess patients in March–June 2020 (multiple answers possible)? | New strategies for assessing EPC patients | - Video consultations - Phone consultations - Chat/messaging consultations - Waiting in their car - Assess patients in their car - No new strategies |
|  |  |  |  |
|  | Did the procedures for responding to emergency calls in the EPC service change because of the pandemic? | New strategies for responding to emergency calls | Yes/ No. If yes, describe |
|  |  |  |  |

* EPC: Emergency Primary Care ** PPE: Personal Protection Equip
